# Central nervous system penetration-effectiveness rank does not reliably predict neurocognitive impairment in HIV-infected individuals

**DOI:** 10.7448/IAS.17.4.19655

**Published:** 2014-11-02

**Authors:** Raffaella Libertone, Patrizia Lorenzini, Pietro Balestra, Carmela Pinnetti, Martina Ricottini, Maria Maddalena Plazzi, Samanta Menichetti, Mauro Zaccarelli, Emanuele Nicastri, Rita Bellagamba, Adriana Ammassari, Andrea Antinori

**Affiliations:** Clinical Department, National Institute for Infectious Diseases Lazzaro Spallanzani IRCCS, Rome, Italy

## Abstract

**Introduction:**

Central nervous system (CNS) penetration-effectiveness (CPE) rank was proposed in 2008 as an estimate of penetration of ARV regimen into the CNS, and validated as predictor of CSF HIV-1 replication. Results on predictive role of CPE on neurocognitive and clinical outcome were conflicting.

**Materials and Methods:**

Retrospective, cross-sectional analysis of neurocognitive profile in HIV-infected cART-treated patients. All patients underwent neuropsychological (NP) assessment by standardized battery of 14 tests on 5 different domains. People were classified as having NCI if they scored >1 standard deviation (SD) below the normal mean in at least two tests, or >2 SD below in one test. Linear and logistic regression analyses were fitted using as outcome Npz8 and impaired/not impaired respectively.

**Results:**

A total of 660 HIV-infected cART-treated individuals from 2009 to 2014, contributing a total of 1003 tests (mean age 49 (IQR 43–56), male 82%; median current CD4 586/mm^3^; 18% HCV infected; HIV-RNA <40 cp/mL in 84%). Current ARV regimen was 2NRTIs+1NNRTI 50.3%, 2NRTI+1PI/r in 32.6%, NRTI sparing in 11.1%. Mean CPE of current regimens was 6.6 (95% CI 6.5–6.7). As per test multivariable analysis, higher CPE values were associated to poor NP tasks (Beta=−0,09; 95% CI −0,14 −0,03; p=0.002 at multivariable linear regression). The association between higher CPE and increased NCI risk was confirmed at multivariable logistic regression, with a 1.24-fold risk of NCI occurrence for each point increase of CPE of current regimen at the time of NP testing (see Table 1). In a sensitivity analysis performed only on patients at the first NP test, the association between higher CPE and poor NP tasks and enhanced NCI risk was only marginally confirmed (Beta=−0,05; [−0,12–0,02]; p=0,19; OR 1,13 [0,95–1,34]; p=0.17). Older age, longer time from HIV diagnosis, current CD4 count <350 cell/mm^3^ and lower education level were all associated to an increased risk of NCI.

**Conclusions:**

In our analysis, higher CPE rank is associated to poorly performing at NP tasking. Even if selection bias could not be excluded due to retrospective cross-sectional design, these results fitted with the direct correlation between high CPE and HIV dementia recently recorded in a large observational database. We think that CPE use to guide ART in patients neurocognitively impaired should be revised.

**Table 1:** Multivariable analysis of predictors of neurocognitive impairment by logistic regression model

Multivariable logistic regression	OR	95%	CI	p
Age (per 10 years incr.)	1.04	1.02	1.05	0.000
Mode of HIV transmission				
Heterosexual	1.00			
Homosexual	1.14	0.81	1.59	0.459
IVDU	0.94	0.51	1.74	0.855
Other/unknown	0.93	0.44	1.95	0.841
Previous aids event	1.72	0.77	3.84	0.186
Years from HIV test (per one year incr.)	1.04	1.02	1.07	0.000
HIV-RNA <40 cp/mL at NPA	0.70	0.45	1.07	0.102
CD4 at NPA, cell/mmc				
> 500	1.00			
350–500	2.20	1.42	3.42	0.000
< 350	1.57	1.07	2.30	0.021
Education (per one year more)	0.83	0.80	0.87	0.000
HCV co-infection				
Negative	1.00			
Positive	1.29	0.82	2.03	0.269
Unknown	1.48	0.76	2.88	0.254
Type of current regimen				
NRTI + NNRTI	1.00			
NRTI + PIB	1.30	0.92	1.85	0.135
NRTI + II	1.70	0.53	5.41	0.369
NTRI sparing	1.09	0.56	2.15	0.792
Other	0.81	0.38	1.76	0.599
CPE 2010	1.23	1.07	1.41	0.003

